# Congenital Infiltrating Lipomatosis of the Face: A Case Report

**DOI:** 10.1155/2012/134646

**Published:** 2012-04-02

**Authors:** Asha Mahadevappa, Vanisri H. Raghavan, Sunila Ravishankar, Gubbanna V. Manjunath

**Affiliations:** Department of Pathology, JSS Medical College, JSS University, Mysore 570 015, Karnataka, India

## Abstract

Congenital infiltrating lipomatosis of the face is a rare lesion that comprises a subgroup of lipomatous tumor-like lesions of infancy and childhood. It is characterized by (1) no encapsulation, (2) diffuse infiltration of mature adipose tissue over normal muscle fiber and surrounding structures of face, (3) osseous hyperplasia of subjacent bone, and (4) a high recurrence rate. We report a case of a nine-month-old infant who presented with swelling over right face since birth. Early diagnosis of this lesion provides better surgical approach to control the infiltrative nature of its growth with recurrence and aesthetic appearance.

## 1. Introduction

Congenital infiltrating lipomatosis of the face (CIL-F) is a distinct clinicopathological rare entity, first described by Slavin et al. in 1983. It comprises a subgroup of lipomatous tumor-like lesions and is characterized by collection of nonencapsulated, mature adipocytes that infiltrate local tissues, leading to craniofacial deformities and tend to recur after surgery. The tumor-like lesion is congenital in origin and occurs in infancy or early childhood as unilateral facial asymmetry [1]. However due to the normal psychomotor development of the children, aesthetic appearance remains the main concern [2]. Till date, fewer than 50 cases have been reported in English literature [3, 4]. We report a case of an infant presenting with unilateral facial hypertrophy since birth.

## 2. Case Report

**Figure 1:**
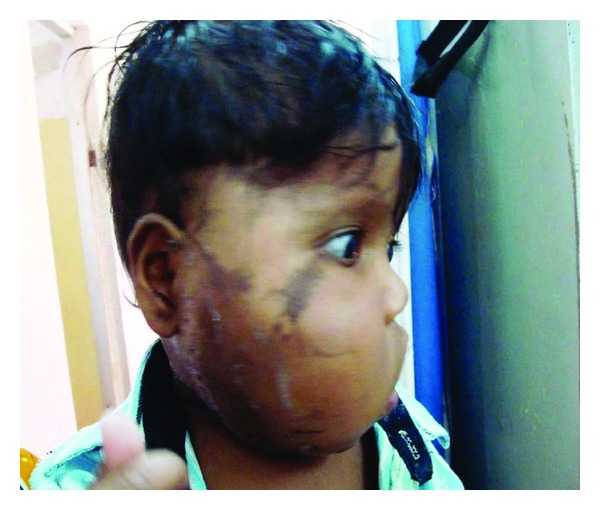
Right diffuse facial swelling.

 A 9-month-old male infant presented with swelling over the right face since birth that had been progressively increasing in size and early eruption of deciduous teeth. Mother gives history of uneventful pregnancy and birth. Developmental mile stones were normal. Local examination revealed a soft nontender, noncompressible, ill-defined, and diffuse swelling over the right side of the face causing unilateral facial hypertrophy (Figure 1). Facial movement, eye closure, and tongue movements were normal with good intake of feeding. No sign of facial nerve compression seen. The computed tomographic (CT) scan revealed moderate-sized diffuse-lipid density lesion in the subcutaneous fat planes over right maxillary, temporal, mandibular area, and mild osseous hyperplasia. Routine hematological investigations were within normal limits. A wide excision of the lesion was performed.

**Figure 2:**
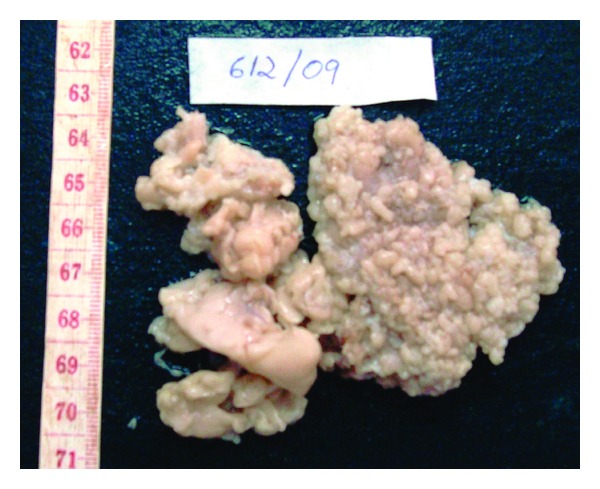
Yellowish white adipose tissue with grey-white areas.

**Figure 3:**
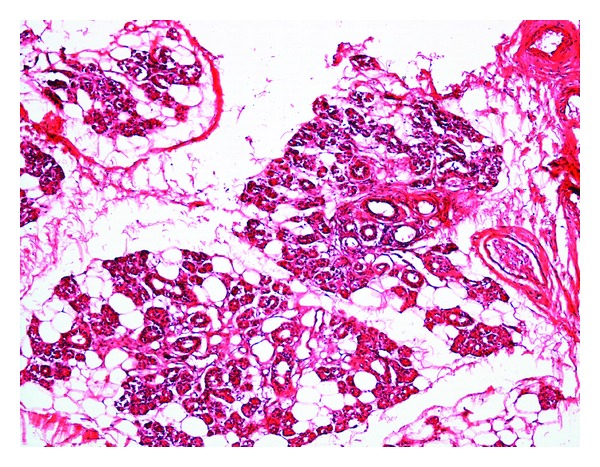
Nonencapsulated, mature adipose tissue infiltrating salivary gland tissue. (H and E, 100x).

**Figure 4:**
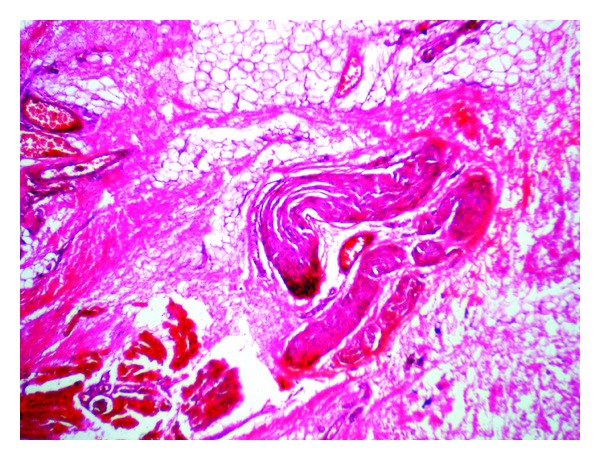
High-power view showing mature adipose tissue infiltrating muscle, nerve fibers, and thickened blood vessels. (H and E, 400x).

 Grossly the specimen measured 8 × 7 cms and showed yellowish white adipose tissue with grey-white areas (Figure 2). Microscopic examination showed nonencapsulated lesion containing mature adipose tissue in lobules. Diffuse infiltration of salivary gland tissue, nerve fibers, skeletal muscle fibers and lymphoid tissue were seen (Figure 3). Presence of fibrous tissue and vessels with thickened wall was noted (Figure 4). Extensive examination showed no nuclear atypia or lipoblasts. A diagnosis of CIL-F was confirmed. The postoperative course was uneventful. There was no facial nerve injury. In the case described postoperative followup for two years has not shown any recurrence. However, patient has been advised for regular followup.

## 3. Discussion

CIL-F is a diffuse overgrowth of fatty tissue of the face, infiltrating local tissues resulting in cranio facial deformity [2]. Earlier studies by Slavin et al. and de Rosa et al. described the following characteristics of CIL-F: (1) nonencapsulated proliferation of mature adipose tissue, (2) diffuse infiltration of muscle and adjacent soft tissue, (3) presence of fibrous tissue, various nerve bundles and vessels with thickened wall, (4) the absence of lipoblasts and signs of malignancy, in spite of a rapid growth rate, (5) hypertrophy of subadjacent bone, and (6) being congenital in origin with a tendency to recur after surgical excision [1, 5].

The facial asymmetry at birth was the common clinical presentation of CIL-F. The other associated clinical features that have been described in the recent studies include ipsilateral hypertrophy of the underlying facial skeleton, increased density of the facial hair on affected side, ipsilateral hemimacroglossia and ptosis, ipsilateral faint cutaneous capillary stain, early eruption of deciduous, permanent teeth and missing teeth associated with dentigerous cyst [4, 6, 7].

Associations with various intracranial abnormalities like hemimegalencephaly, arachnoid cyst [8], and mucosal neuromas [2, 6] have been described. In the case described, no intracranial abnormalities noted. Heymans and Ronsmans in their study concluded that the origin of all the deformities observed in CIL-F would be neuronal crest anomalies [2]. The etiopathogenesis of CIL-F is unclear and biological behavior remains uncertain [4, 7, 9]. However, the possible mechanisms for the lipomatous change have been proposed are trauma, chronic irradiation, muscularmetaplasia, degenerative processes with fatty transformation, multipotential cells of embryogenic origin under the influence of hormones, congenital cytomegalovirus infection, and alteration in chromosome 12 [1, 5, 9].

Microscopically, this lesion has to be differentiated from various lipomatous lesions. CIL-F is termed lipomatosis because it is more diffusely infiltrative in nature than a lipoma, which is well defined and encapsulated. Lipoblastomatosis is composed of immature lipoblasts and most occur on the extremities, trunk, and neck. Liposarcomas differs from CIL-F by the presence of pleomorphism, mitotic figures with capillary network and are usually located on the trunk and extremities, and rarely occur in infantile or congenital forms [2, 9]. The final distinction among these lipomatous lesions requires histopathological confirmation [8]. CT scan and magnetic resonance imaging** (**MRI) identify lipo density and provide better delineation of the extent of the lesion and characterization of CIL-F [3, 8, 9]. Clinically the different lesions causing unilateral facial enlargement include infections, trauma, vascular or lymphatic malformations, benign and malignant tumours of soft and hard tissues. Also considered are the possibilities of hamartomatous or overgrowth syndromes such as proteus syndrome, encephalocutaneous lipomatosis, Cowden syndrome, and Bannyan-Riley-Ruvalcava syndrome [4, 8, 9]. Clinical examination with MRI and histopathological findings helps in the diagnosis of CIL-F.

The treatment modalities available are excision or liposuction mainly for cosmetic reasons [2, 3, 8, 9]. The type of surgical treatment depends on the extent and clinical findings of the lesion. Due to its diffuse infiltration and involvement of important facial structures, complete surgical excision is often impossible and results in high recurrence rate after surgical intervention [2, 4, 9]. Progression of CIL-F varies from extensive hypertrophy occurring in early childhood to a more indolent form with hypertrophy occurring over decades, with patients presenting for treatment in adulthood [4, 5]. There is no report of malignant change over 2–14-year followup [1, 2]. Hence, surgical treatment improves the aesthetic appearance of each child despite the lesion persistence and recurrence [1, 2, 6].

## 4. Conclusion

CIL-F of the face is a rare benign disorder of lipomatous tissue in infancy or childhood. It should be considered when evaluating the cause of facial asymmetry. Clinical examination, imaging studies, and histopathological findings help in early diagnosis. The main purpose of surgery is to improve the cosmetic appearance of the face rather than to eradicate the tumour.
